# Australia for the Sons of Professional Men

**Published:** 1909-02-13

**Authors:** Richard Arthur

**Affiliations:** M.D. President, Immigration League of Australasia. Sydney


(*To the Editor of* The Hospital.)

### AUSTRALIA FOR THE SONS OF PROFESSIONAL MEN.

*To the Editor of* The Hospital.

Sir,—Since my letters dealing with the opportunities offered by Australia to the sons of professional men have appeared in your columns, I have received a large number of communications from doctors, lawyers, chemists, etc., who are anxious to send their sons to Australia and enter them at one of the agricultural colleges here or get them straight on to a farm. So great has this response been that I have decided to attend the next annual meeting of the British Medical Association at Belfast and seek there an opportunity of bringing the advantages of land settlement in Australia before the members of the Association who may be interested in the subject. I hope to be in London by May, and letters addressed to me at the Royal Colonial Institute, Northumberland Avenue, W.C., will be promptly attended to. In the meantime, illustrated prospectuses of the various agricultural colleges can be obtained by application to Captain Collins, Commonwealth Offices, Victoria Street, S.W., and if any gentlemen decide to send their sons out at once so as to arrive here in April or May, which is a favourable time of the year for doing so, I should be much obliged if they would notify this to the Secretary of the Immigration League of Australasia, 14 Moore Street, Sydney. At several of the colleges in New South Wales the fees for board and tuition are to be reduced to £10 or £12 per annum, which should bring this invaluable course of agricultural training within the reach of everyone. I should again like to emphasise the fact that the accommodation and food at these institutions are excellent, and that a thorough scientific and practical education in all the branches of farming, dairying, stock-raising, and fruit-growing is given. But a perusal of one of the prospectuses will be far more convincing with regard to this than anything I can write. And I am certain that once their sons are settled in Australia, many professional men will seriously consider the idea of following them out here, and of spending the remainder of their days in the glorious climate of this country, where judicious investment of money in town and country lands can often double and treble one's capital in a few years.

Yours etc.,

Richard Arthur, M.D.

President, Immigration League of Australasia.

Sydney, December 28.

